# Supplementary material for: In Model, In Vitro and In Vivo Killing Efficacy of Antitumor Peptide RDP22 on MUG-Mel2, a Patient Derived Cell Line of an Aggressive Melanoma Metastasis
Source: Biomedicines. 2022 Nov 17;10(11):2961. doi: 10.3390/biomedicines10112961 (PMC9687695; doi:10.3390/biomedicines10112961)
Supplement: Supplementary file 1 [file biomedicines-10-02961-s001.zip › biomedicines-1958123-supplementary/Figure S1.pdf]

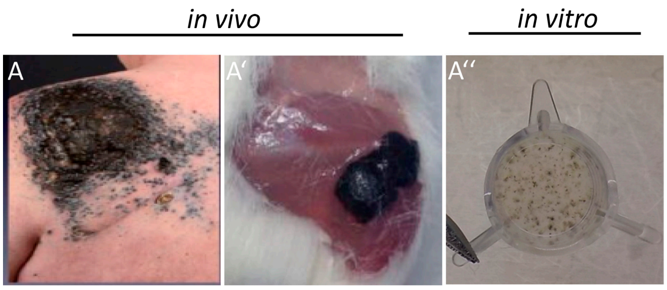

Figure S1. Persisting pigmentation of MUG-Mel2 in vivo and in vitro. A) Cutanous metastases of a male patient as source for the MUG-Mel2 cell line. (A′) Black tumor growing within 10 days in NSG mouse. (A′′) Human 3D in vitro MUG-Mel2 melanoma model (mOS-REpMUG-Mel2) with pigmented melanoma clusters.
