# Supplementary material for: In Model, In Vitro and In Vivo Killing Efficacy of Antitumor Peptide RDP22 on MUG-Mel2, a Patient Derived Cell Line of an Aggressive Melanoma Metastasis
Source: Biomedicines. 2022 Nov 17;10(11):2961. doi: 10.3390/biomedicines10112961 (PMC9687695; doi:10.3390/biomedicines10112961)
Supplement: Supplementary file 1 [file biomedicines-10-02961-s001.zip › biomedicines-1958123-supplementary/Figure S2.pdf]

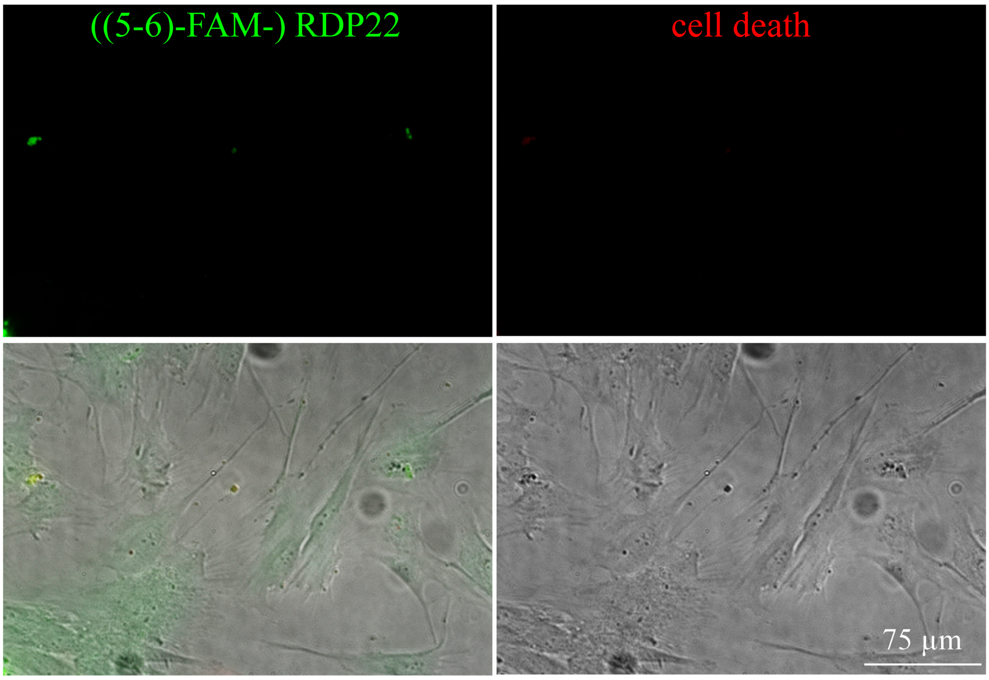

Figure S2. Interaction of RDP22 with non-malignant control cells. 10 µM fluorescently labeled ((5–6)-FAM-) RDP22 (green) was incubated with non-malignant control cells of normal human dermal fibroblasts (NHDF) for 4 hours in presence of PI (red). In the second-row overlaps of fluorescence channels and bright field, respectively solely bright field pictures are shown. The pictures represent outcome of 3 independent data sets. RDP22 shows no significant interaction (green) or significant cell death (red) with or within NHDF cells.
