# Supplementary material for: In Model, In Vitro and In Vivo Killing Efficacy of Antitumor Peptide RDP22 on MUG-Mel2, a Patient Derived Cell Line of an Aggressive Melanoma Metastasis
Source: Biomedicines. 2022 Nov 17;10(11):2961. doi: 10.3390/biomedicines10112961 (PMC9687695; doi:10.3390/biomedicines10112961)
Supplement: Supplementary file 1 [file biomedicines-10-02961-s001.zip › biomedicines-1958123-supplementary/Figure S3.pdf]

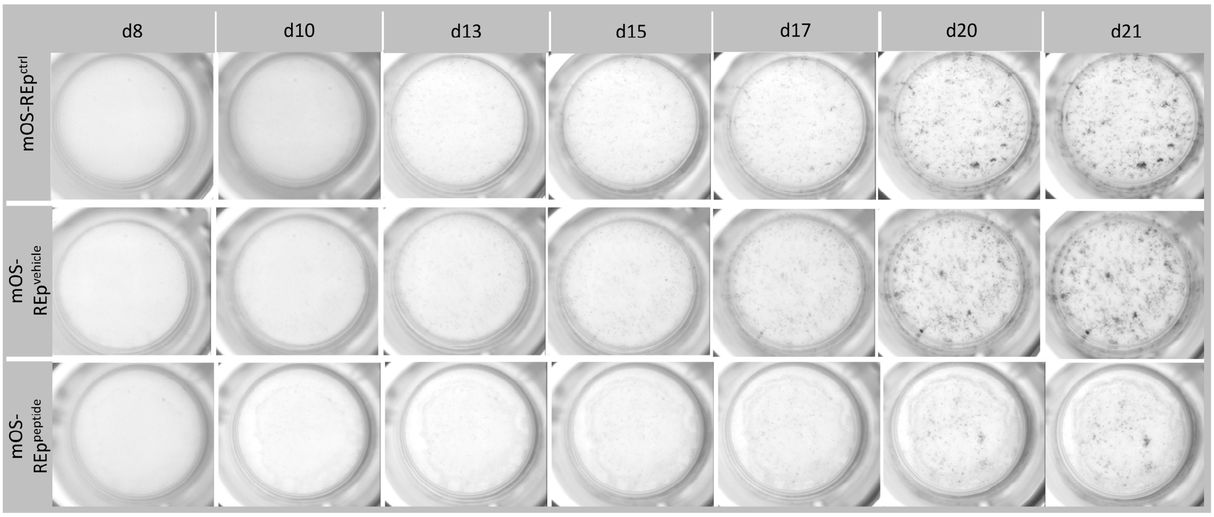

Figure S3. Non-invasive fully automated detection of melanoma pro- and regression. Images were taken during 21 days of culture duration at defined time points (d8, d10, d13, d15, d17, d20 and d21) using the in-house developed MediTOM device.
